# Methods, markers and mechanisms for protective immunity in the controlled human malaria infection model

**DOI:** 10.1186/1475-2875-13-S1-O32

**Published:** 2014-09-22

**Authors:** Robert Sauerwein

**Affiliations:** 1 Radboud University Medical Center, Nijmegen, The Netherlands

## 

A unique tool to study malaria immunology and efficacy of immunization strategies form Controlled Human Malaria Infections (CHMI) and has proved to be a reproducible, predictable and safe method of inducing *Plasmodium falciparum* (Pf ) malaria. An efficient method for induction of complete protection in humans was achieved by us in a CHMI setting by exposing human subjects to Pf-infected mosquitoes while taking blood-stage suppressive chloroquine prophylaxis. When tested in clinical trials, this protocol induced clinical and parasitological protection against a standard homologous and heterologous CHMI. In addition, we showed that CPS-induced protection was long lasting and primarily mediated by immunity to sporozoite and liver stages rather than to asexual blood-stages. Cellular responses to *Plasmodium falciparum* parasites, in particular interferon-gamma (IFNγ) production, CD107a CD4 cells and Granzyme producing CD8 cells, play an important role in anti-malarial immunity.

Our approach appears to utilize the Pf parasite’s clinically salient replicative phase of liver stage development to induce fully protective immune response against sporozoites and liver stages. It opens opportunities to explore mechanisms of protective immunity, allowing the search for immune correlates/signatures of protection and clinical development of a whole sporozoite based vaccine.

